# Analysis of Associated Spinal Fractures in Cases of Traumatic Intracranial Hemorrhage or Skull Fracture

**DOI:** 10.5704/MOJ.1603.003

**Published:** 2016-03

**Authors:** M Yunoki, K Suzuki, A Uneda, K Yoshino

**Affiliations:** Department of Orthopaedics, Kagawa Rosai Hospital, Marugame City, Japan

**Keywords:** Fracture, skull, spine, intractranial hemorrhage

## Abstract

**Introduction:** Patients with traumatic intracranial hemorrhage (ICH) or skull fracture are typically admitted to the Department of Neurosurgery for fear of delayed neurological deterioration. Neurosurgeons, therefore, must be careful not to overlook a spinal fracture in these patients. In this study, we investigated the occurrence and risk factor of spinal fracture in patients with traumatic ICH or skull fracture.

**Patients and methods:** We retrospectively analyzed the hospital records of 134 patients admitted to the Department of Neurosurgery at Kagawa Rosai Hospital for traumatic ICH or skull fracture. The etiology of trauma, level of consciousness, presence or absence of ICH, skull fracture, craniotomy and spinal surgery were investigated. Furthermore, in cases of spinal fracture, its type, neurological symptoms, treatment were investigated.

**Results:** In an analysis of 134 patients, Ground level fall and traffic accident were the most frequent etiologies of trauma (47.0% and 23.9% respectively). Glasgow coma scale on admission was 15-13 for 106 patients (79.1%). spinal fracture was identified in 10 of 134 patients (7.5%). Two patients had cervical, 8 had thoracolumbar fractures. In the analysis of risk factors, an accidental fall and skull fracture was observed significantly more in the spinal fracture cases.

**Conclusion:** The majority of traumatic ICH or skull fracture cases treated in the Department of Neurosurgery were caused by minor head impacts. When treating these patients, it is necessary to investigate not only the cervical, but also the thoracolumbar spine, especially when the cause of injury is an accidental fall and a skull fracture is identified.

## Introduction

In Japan, there are many facilities where trauma patients, whose main injury site is the head, are treated predominately by neurosurgeons. Even when head trauma is accompanied by an intracranial hemorrhage (ICH) or skull fracture, patients with even mild symptoms are admitted directly to the Department of Neurosurgery for fear of delayed deterioration^1^. Neurosurgeons, therefore, must be careful not to overlook spinal fracture in these patients.

The association between head and cervical spine injuries has long been recognized. The reported incidence of cervical spine trauma after a clinically significant head injury ranges from 4-8%^2^. Moreover some authors have reported that an increase in injury severity is associated with a higher rate of cervical injury^3^. However, little is known about the risk of spinal fracture in trauma patients who are routinely admitted to the Department of Neurosurgery.

In the present study, we performed an analysis to determine the incidence and characteristics of spinal fractures in patients who sustained a traumatic ICH or skull fracture. We also sought to identify the risk factors for spinal fracture to more effectively identify high risk patients at the early stage of their hospitalization.

## Materials and Methods

For the trauma patients in our hospital, in cases where an ICH or skull fracture is identified in a head computed tomography (CT), cervical radiology or cervical spine CT is performed as a general rule. In case of high energy trauma or if the patient complains of back pain, a CT scan to evaluate the thoracolumbar spine is additionally performed. In this study, 134 patients admitted to our department from October 2012 to December 2014 for traumatic ICH or skull fracture without a high level of damage of other organs were evaluated retrospectively. In all cases, the etiology of trauma, Glasgow coma scale (GCS), presence or absence of ICH, skull fracture and vertebral fracture were investigated retrospectively. For cases of vertebral fracture, we investigated the type of fracture, neurological symptoms, and treatment status.

The etiology of trauma was classified into four groups: ground level fall (GLF), accidental fall, traffic accident and others. Among the accidental falls, a fall from less than 2 m was defined as a fall from a low height, more than 2 m was defined as a fall from a high height. Furthermore, the cause of a traffic accident was classified into four groups according to whether the patient was a cyclist, car driver, motorcyclist or pedestrian. ICH was classified into four groups: subarachnoid hemorrhage (SAH), subdural hematoma (SDH), epidural hematoma (EDH) and contusion hematoma (CH). The extent of spinal injury was defined by the American Spinal Injury Association Impairment Scale (AIS).

Data were expressed as the mean ± standard deviation (SD). For two-group comparison of a categorical variables chi-square test or Fisher’s Exact Test was used. To compare mean ages of two-groups, Student’s t-test was used. Variables were considered to be statistically significant when their significance levels were <0.05.

## Results

### Analysis of patients with traumatic ICH or skull fracture

Characteristics of patients with traumatic ICH or skull fracture are shown in Table I. The mean age was 66.6 ± 21.0 years (male:female = 92:42 ). GLF (63 cases; 47.0%) was the most common mechanism, followed by accidental fall (26 cases; 19.6%). The GCS on admission was 15-13 in 88 cases (79.1%), followed by 8-3 in 17 cases (12.7%), 12-9 in 11 cases (8.2%). Emergency craniotomy was performed in 13 cases (9.7%) and 14 patients died within one month (10.4%).

**Table I:**
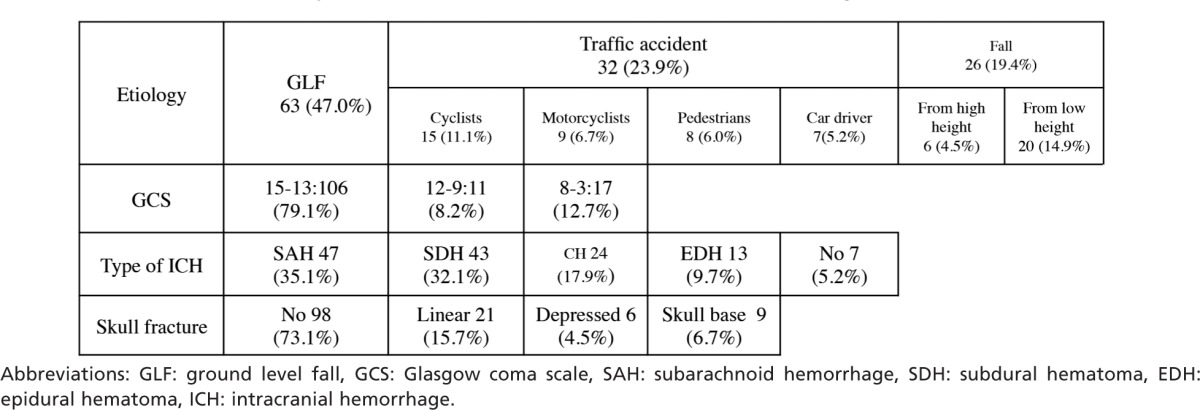
Summary of 134 patients with traumatic intracranial hemorrhage or skull fracture

### Analysis of patients with spine fracture

Of the 134 cases with traumatic ICH or skull fracture, a spinal fracture was confirmed in ten cases (7.5%). The mean age of these patients was 64.8±21.3 years (male:female = 7:3). Of the ten patients, two had cervical, four each of thoracic and lumbar fractures. Eight of these patients were classified as AIS E. In four out of ten, the spinal fracture was identified at the time of admission. In the other six cases, the spinal fracture was diagnosed five hours to two months after admission. In these cases, spinal CT was performed to explore the cause of persistent back pain or vertebral artery occlusion. Spinal surgery was performed for six patients (60%). Posterior fixation was carried out without major complication for these six patients.

An accidental fall (six cases, 60%; four cases were from a low height, two cases were from a high height) was the most common mechanism, followed by traffic accident (three cases, 30%: two were car drivers, and one was a cyclist). The GCS was 15 in seven cases (70%). Regarding the type of ICH, three cases (30%) of SAH and EDH, two cases (20%) of SDH and one case (10%) of CH were recognized. Craniotomy was performed in two cases to remove SDH and EDH. Skull fracture was identified in six cases. The most common type of skull fracture was a linear fracture (four cases; 40%) followed by a depressed fracture (one case; 10%) and skull base fracture (one case; 10%) (Table II). It was confirmed that all ten of the spinal fracture patients survived for more than one month.

**Table II:** Summary of 10 patients of spinal fracture with traumatic intracranial hemorrhage or skull fracture

	Age/sex	Etiology	Type of ICH	Type of skull fx	GCS	Spinal fracture		Diagnosis of spinal fx after admission	AIS	Cranio tomy	Spinal surgery
						**Level**	**Type**				
1	80/M	Fall from low height	SAH	Skull base	3	C4/5	Transdisca l	5 hours	A	-	Posterior fixation
2	26/M	Car driver	-	Linear	15	L2-5	transverse process	On admission	E	-	-
3	39/M	Fall from low height	SAH	-	15	Th12	burst	On admission	E	-	Posterior fixation
4	75/M	Fall from low height	EDH	Linear	15	L1	burst	2 days	E	-	Posterior fixation
5	78/F	GLF	SAH	-	15	C2	Type2 (Anderson)	5 days	E	-	Magerl
6	84/F	cyclists	EDH	Linear	15	L1	compression	12 days	E	-	-
7	70/M	Fall from high height	EDH	Linear	9	L1	burst	On admission	E	+	Posterior fixation
8	75/F	Fall from low height	SDH	-	15	Th7	compression	5 days	E	-	-
9	86/M	Fall from high height	SDH	Depresse d	6	Th11	burst	On admission	A	+	Posterior fixation
10	35/M	Car driver	ICH	-	13	Th2-5	Spinous process	2 Mo	E	-	-

Abbreviations: AIS: the American Spinal Injury Association classification, fx: fracture, ICH: intracranial hemorrhage

### The analysis of the risk factors associated with spinal fractures

There was no significant difference in the mean age of the patients with and without spinal fractures (64.8±21.3 years vs 65.5±21.3 years, respectively; p=0.71). Because seven of the ten patients with spinal fractures were over 70 years of age, we also performed a comparison of two age groups: patients who were below70 years of age and those who were above 70 years of age. This also failed to show significant difference (OR = 1.62, 95% CI [0.35, 10.1]). A categorical comparison was performed with sex, injury mechanism, GCS category and the presence of skull fracture, ICH and craniotomy included as potential risk factors. No significant differences were detected in sex, GCS category or the presence of ICH and craniotomy. In the analysis of the injury mechanisms, patients with a traumatic fall were significantly more likely to suffer a spinal fracture than those with a non-traumatic fall (OR = 7.6, 95% CI [1.60, 40.2]). A significant difference was also recognized between the patients with and without a skull fracture (OR = 4.6, 95% CI [1.60, 40.2] (Table III). The odds of a cervical spine injury with various injury mechanisms and in patients with a skull fracture were also analyzed. In spite of the limited sample size, a fall from high height tended to be associated with a greater likelihood of spinal fracture in comparison to the various injury mechanisms that were analyzed. The odds of a spinal fracture did not differ among the different types of skull fracture. (Table IV)

**Table III:** A univariate analysis of the predictors of spinal fracture that were associated with traumatic intracranial hemorrhage or skull fracture

		Spinal fracture				
		**Present (n=10)**	**Absent (n=124)**	**Odds ratio**	**95%CI**	**P value**
Sex	Female	3	39	1.0		
	Male	7	85	1.9	0.36–19.1	P=0.72
Age group	<70	3	55	1.0		
	≥70	7	79	1.6	0.35–10.1	P=0.74
Mechanism	Non–accidental fall	4	104	1.0		
	Accidental fall	6	20	7.6	1.60–40.2	P<0.05
GCS	15–13	6	100	1.0		
	12–9	2	9	0.3	0.04–3.17	P=0.17
	8–3	2	15	2.2	0.20–13.9	p=0.31
Intracranial hemorrhage	Absent	1	6	1.0		
	Present	9	118	0.5	0.05–23.4	P=0.43
Skull fracture	Absent	4	94	1.0		
	Present	6	30	4.6	1.02–23.9	P<0.05
Craniotomy	+	2	8	1.0		
	–	8	116	3.57	0.32–22.8	P=0.16

Abbreviations: CI: confidence interval, GCS: Glasgow coma scale

**Table IV:** The odds of a cervical spine injury with each of the injury mechanisms and in patients with skull fracture

			Spinal fracture				
			Present (n=10)	Absent (n=124)	Odds ratio	95%CI	P value
Mechanism	Fall	High height	2	4	1.0		
		Low height	4	20	0.41	0.04–6.06	P=0.57
	Traffic	Pedestrian	1	8	0.53	0.004–6.82	P=0.53
	accident	Car driver	2	7	0.59	0.03–11.3	P=1.0
	GLF		1	62	0.04	0.001–0.84	P<0.05
Skull fracture	Linear		4	17	1.0		
	Depressed		1	6	1.40	0.11–80.9	P=1.0
	Skull base		1	8	2.2	0.20–13.9	p=1.0

Abbreviations: CI: confidence interval, GLF: ground level fall

## Discussion

In this study, spinal fracture was identified in ten of 134 cases with traumatic ICH or skull fracture (7.5%). Of the ten cases, eight cases of lumbar or thoracic fracture were recognized (6.0%), but only two cases of cervical spine fracture were identified (1.5%). The incidence of cervical spine trauma after head injury has generally been reported to range from 4 to 8%^2^. Moreover, Holly *et al* (2002) analyzed 447 patients with moderate and severe head injury (238 with an initial GCS score of 8 and 209 with an initial GCS score of 9 - 14) and concluded that patients with an initial GCS score of less than or equal to eight were more likely to sustain a cervical injury than those with a score higher than eight^3^. In our study, GLF was the most common mechanism (63 cases; 47.0%). The GCS on admission was 15-13 in 106 cases (79.1%), which means that the majority of our patients experienced a low impact. Our study was associated with a limitation in the small number of patients with moderate and severe head trauma, which may be the reason why the incidence of cervical spinal fractures was lower than that reported in other studies.

As for thoracolumbar spinal fracture, there are a few reports the incidence in head trauma patients. Paiva *et al.* (2011) analyzed 180 patients with moderate or severe head injuries (GCS of 3 - 12)^4^. They identified spinal cord injury in 14 patients (7.8%), 12 were located in the cervical spine, one was in the lumbar spine and one was in the thoracic spine. The most common causes of brain trauma were pedestrians being struck by motor vehicles (31.1%), car crashes (27.7%), and falls (25%). Their study was also based on more severe head injury and the mechanisms were quite different from those in our study. Some epidemiological studies reported that the most frequent site of spinal fractures is the thoracolumbar transition, and the most frequent cause of which is an accidental fall^5^. Because most of the cases of traumatic ICH or skull fracture that were treated in our department were caused by a low impact head injury, the results of our study were similar to such epidemiological studies. As previously mentioned, a CT scan for the evaluation of the thoracolumbar spine was included in the case of a high energy trauma in the patient who complained of back pain. However, four out of eight thoracolumbar spine fractures were diagnosed after hospitalization (Table II). In these cases, thoracolumbar CT was not performed before hospitalization because the trauma was low energy and the patients did not complain of severe back pain. When the patients became ambulatory, however, their back pain became apparent and thoracolumbar CT was performed, which revealed the spinal fracture. Even in cases of low-energy trauma, the presence or absence of back pain must be confirmed when the patient becomes ambulatory.

In Case 1, we did not pay attention to the presence of ankylosing spinal hyperostosis (ASH) on arrival, which caused a delay in the diagnosis of the spinal fracture. ASH is diagnosed when flowing ossification of the anterior longitudinal ligament is present on spine radiographs over at least four consecutive levels^6^. Its etiology is unknown, however, associations with obesity, type 2 diabetes mellitus and advanced age have been demonstrated in several groups^6,7^. ASH is generally asymptomatic, but patients are prone to fracture after minor trauma^7^. Moreover, ASH may raise difficult diagnostic issues such as in Case 1. ASH-related spine injuries may become a rapidly increasing condition in modern affluent societies^7^, and awareness of this condition should therefore be raised among physicians assessing head trauma patients.

Fracture of the spinous process is known to be stable but painful. Generally, this type of fracture is treated conservatively without the need for surgical intervention^8^. However, an isolated spinous process fracture is considered to be a warning sign for more severe spinal injury; thus it should be carefully evaluated to detect more severe spinal injuries. In Case 10, a fracture of the spinous process was not detected until two months after trauma because an initial cervical CT scan did not include the Th2-4 spinous process. The frequent sites of spinous process fractures are reportedly the lower cervical and upper thoracic spine^9^. This site is the apex of kyphosis, where the spinous process is relatively thin and projected horizontally. Consequently, excessive tension generated in the supra and interspinous ligament produced by cervical hyperflexion tends to cause spinous process fracture. When a patient complains of upper thoracic or cervical back pain, a radiographic evaluation is necessary to confirm if a spinal process fracture is present. Fracture of the transverse process in the lumbar spine is also known to be a frequent and stable injury^10^. However, lumbar transverse spinous process fractures are rarely complicated by lumbar artery damage, which can cause crisis of life and necessitates rapid performance of TAE^11^. Thoracolumbar CT, therefore, should be performed if the vital signs deteriorate after hospitalization.

In the initial management of head injury patients, the diagnosis of spinal fracture is difficult due to an altered level of consciousness. It is necessary to perform spinal CT, including the thoracolumbar region, at the initial visit especially when the injury etiology is an accidental fall.

## Conclusion

In cases of traumatic ICH or skull fracture, particularly when the cause of injury is an accidental fall and accompanying skull fracture, it is necessary to investigate not only the cervical, but also the thoracolumbar spine.
